# Microbial diversity of meat products under spoilage and its controlling approaches

**DOI:** 10.3389/fnut.2022.1078201

**Published:** 2022-12-01

**Authors:** Yanli Zhu, Wei Wang, Ming Li, Jiamin Zhang, Lili Ji, Zhiping Zhao, Rui Zhang, Demin Cai, Lin Chen

**Affiliations:** ^1^Key Lab of Meat Processing of Sichuan Province, Chengdu University, Chengdu, China; ^2^College of Animal Science and Technology, Yangzhou University, Yangzhou, China

**Keywords:** livestock, poultry, fish meat, microbial spoilage, bacterial community

## Abstract

Meat spoilage (MS) is a complex microbial ecological process involving multiple specific microbial interactions. MS is detrimental to people's health and leads to the waste of meat products which caused huge losses during production, storage, transportation, and marketing. A thorough understanding of microorganisms related to MS and their controlling approaches is a necessary prerequisite for delaying the occurrence of MS and developing new methods and strategies for meat product preservation. This mini-review summarizes the diversity of spoilage microorganisms in livestock, poultry, and fish meat, and the approaches to inhibit MS. This would facilitate the targeted development of technologies against MS, to extend meat's shelf life, and effectively diminish food waste and economic losses.

## Introduction

According to a report by the Food and Agriculture Organization of the United Nations, one-third of food produced for human consumption is either spoiled or wasted (1). MS is defined as a change in color and the production of off-flavors, mucus, and exudates that result in unacceptable sensory and organoleptic properties. Parlapani confirms that the deterioration is caused by specific spoilage organisms that dominate and form metabolites that alter the organoleptic properties of the meat, making it unfit for consumption (2). Although the causes for meat deterioration vary, bacteria direct the process more than other factors such as endogenous enzymes. Meat is generally considered sterile before slaughter, but the environment during slaughter is not sterile, so some degree of microbial contamination may occur, leading to meat corruption (3). The sources of microbial contamination in this process can be summarized as both endogenous and exogenous. The microbiological quality of post-slaughter meat depends to a large extent on the type of meat, processing, distribution, and storage conditions. Contaminated slaughter equipment, personnel and environmental factors (e.g. water, air, and soil) can be cross-contaminated with spoilage-associated bacteria (4). After storage, various intrinsic and extrinsic factors affect the process of microbial MS, including oxygen demand, pH, temperature, and competing organisms (5). The diversity of these ecophysiological factors affects the dynamics of microbial growth, including microbial succession and microbiota composition, ultimately affecting the type and rate of MS. Several strategies have been proposed to preserve fresh products to overcome MS, including the addition of ingredients such as food preservatives, essential oils and storage under refrigerated conditions, and aeration packaging (6, 7). Therefore, understanding the sources of spoilage microorganisms in meat, the diversity of microorganisms and measures to retard spoilage, and achieving accurate and effective inhibition of spoilage microorganisms is one of the common goals of meat industry sessions and academia.

## Spoilage microbial diversity

### Major types of spoilage microorganisms in livestock meat

Not all bacteria cause spoilage of food, there is only an initial small group of microorganisms in meat, referred to as specific spoilage organisms (SSO) (8). In meat products, SSO metabolizes available substrates during storage, leading to changes in meat quality and odor (9). This section summarizes the common microorganisms and spoilage phenotypes associated with the spoilage of livestock meat (Table 1). A study showed that the dominant bacteria in the meatballs of the blown pack spoilage (BPS) group packed with 71.85% CO_2_ were *Klebsiella* (46.05%) and *Escherichia* (39.96%). *Klebsiella pneumoniae* was the main strain causing BPS in meatballs due to its ability to pack swelling (26). Wang et al. (27) revealed that *Proteobacteria, Firmicutes, Pseudomonas spp*., *Acinetobacter spp*., *Pantoea spp*., *Brochothrix spp*., and *Raoultella spp*. were the main pathogenic and spoilage bacteria in chilled pork by culture-dependent and non-culture-dependent methods (27). The microbial composition of pork stored at−2°C and 4°C showed a high degree of similarity, with *Pseudomonads* and *Brochothrix* being the dominant taxa. *Acinetobacter spp*., *Myroides spp*., and *Kurthia spp*. were markers for spoiled pork meat stored at 25°C (28). The current research results show that the abnormal growth of lactic acid bacteria, *Micrococcaceae, Enterobacteriaceae*, yeast, and mold plays a key role in the formation of dry cured ham odor defects, while the key putrefactive microorganisms of different types of ham are different (29, 30). In Mianning ham, the dominant bacterial genus was *Clostridium_sensu_stricto_*2 (92.01%), and the dominant fungal genus was *Aspergillus* (84.27%) (31). The number of *Enterobacteriaceae* and *Enterococcus* in deteriorated ham was significantly higher than that in normal ham. High water content and low salt content lead to abnormal growth of *Enterobacteriaceae* and *Enterococcus* in deteriorated ham, leading to the deterioration of Jinhua ham (32, 33). *C. farmei CDC 2991–81, B. cereus ATCC 14579*, and *E. faecalis ATCC 19433* were the main spoilage microorganisms of Jinhua ham (34). *C. sestertheticum* was detected as the most abundant *Clostridium spp*. in vacuum packaging beef and other raw meats, associated with BPS (35). Li et al. (36) reported that total viable bacteria (8.75 log CFU/cm^2^) and Lactobacillus (3.20 log CFU/cm^2^) counts were higher on meat surfaces dry-aged for 19 days (36). The microbial communities of all samples evaluated in dry-aged beef contain *Enterobacteriaceae* and *Pseudomonas*, which are considered to be the major spoilers in dry-aged beef (13). In another study, beef and lamb samples from Europe, North and South America, and Oceania were investigated and *Psychrophilic Clostridium spp*. was found to be the most prevalent *Clostridium* (37).

**Table 1:** List of bacterial groups associated with livestock and poultry MS.

**NO**.	**Bacteria genus**	**Spoilage phenotype**	**References**
**Livestock**		
1	LAB	Slime and discoloration	(8)
2	*Leuconostoc*	Slime, gas production and discoloration	(9, 10)
3	*Lactobacillus*	Slime	(9, 11)
4	*Serratia*	Discoloration	(12)
5	*Weissella*	Slime and Discoloration	(11)
6	*Enterobacteriales*	Slime and discoloration	(13)
7	*Clostridium*	Blown pack spoilage	(14, 15)
8	*Pseudomonas*	Biofilm formation	(16)
9	*Brochothrix*	Biofilm formation	(17)
**Poultry**	
1	*Aeromonas*	Biofilm formation	(18, 19)
2	*Salmonell*	Unknown	(20)
3	*Pseudomonas*	Slime formation and meat softening	(21)
4	*Enterococcus*	Discoloration	(22)
5	*Brochothrix*	Discoloration	(41)
6	*Acinetobacter*	Biofilm formation	(23)
7	*Shewanella*	Discoloration	(24)
8	*Staphylococcus*	Acid production	(25)

### Major types of spoilage microorganisms in poultry meat

There has been a steady increase in consumption and demand for poultry meat globally. Among poultry products, processed chicken meat is the most consumed (about 75% of total poultry meat), followed by turkey (about 25%) and duck meat (38). The bacterial community in poultry meat include pathogenic species such as *Salmonella* and *Campylobacter* (18). This section summarizes the common microorganisms and spoilage phenotypes associated with the spoilage of poultry meat (Table 1). When defining the dominant spoilage bacteria in the spoilage process of meat products based on the number of bacteria, *Pseudomonas spp*., *Bacillus spp*., *Crude Typhimurium spp*., *Schwartzella spp*., *Aeromonas spp*. are usually considered to be the dominant communities in cold meat and poultry packed under aerobic conditions (19). Poultry meat spoils quickly, even under refrigerated conditions. Wang et al. detected a significant increase of *Clostridium* perfringens over time in almost poultry samples stored aerobically under different refrigeration conditions. *Pseudomonas fluorescens, Aeromonas salmonicida*, and *Serratia liquefaciens* cause spoilage of poultry meat stored at 8°C for 4 days (20). Several new enterococci or lactic acid bacteria were also identified in poultry products, such as *Viikkiensis enterococcus, Seigonensis enterococcus*, and *Heterofermentative lactic acid bacteria* (39, 40).

With the development of MS studies, it is more appropriate to determine the dominant spoilage organism by determining the spoilage capacity of bacterial isolates grown *in situ*. The main common *Pseudomonas* in poultry meat is *Pseudomonas fragilis, Pseudomonas lundengensis*, and *Pseudomonas fluorescens*. *Pseudomonas fragilis, Pseudomonas fluorescens*, and *Pseudomonas aeruginosa* produced slime on meat and its products during storage (41). Extracellular enzymes secreted by *Pseudomonas aeruginosa* have strong protease activity against myogenic fibronectin and myxomatosis protein. This helps bacteria penetrate the meat to obtain new sources of nutrients, increasing the formation of mucus and softening the meat (21). In addition, *Serratia spp*., *Micrococcus spp*., *Serratia spp*., and *Brucella spp*. were also associated with slime production and softening during MS (42).

### Major types of spoilage microorganisms in fish meat

The increase in the global population has led to an increase in the consumption of fish and meat in various countries. It becomes highly susceptible to spoilage through a series of chemical reactions, under the action of microorganisms and enzymes due to its high water content and high pH (43). Similar to other meat, not all microorganisms in fish meat have the potential for corruption, except SSO. This section summarizes the common spoilage microorganisms associated with the spoilage of fish meat (Table 2).

**Table 2:** List of bacterial groups associated with fish meat spoilage.

**NO**.	**Bacteria genus**	**Fish meat**	**References**
1	*Shewanella*	Raw lobster tails, whole lobster, salmon	(44, 45)
2	*Pseudomonas*	Raw lobster tails, whole lobster, salmon, carp	(44)
3	*Photobacteriu*	Salmon	(44, 46)
4	*Staphylococcus*	Fresh shrimp	(47)
5	*Psychrobacter*	Whole lobster, cod	(48)
6	*Brochothrix*	Raw salmon, catfish, sea bass, sea bream	(48)
7	*B.Thermospheracta*	Fresh shrimp	(49)
8	*Serratia*	Oysters,fish, tropical shrimps	(50)
9	*Salmonicida*	Shrimps, salmon, sea beam	(51)

Møretrø et al. (44) found higher levels of *Pseudomonas spp*. and *Salmonella spp*. on industrially processed salmon filets with the methods of bacterial enumeration 16S rRNA analysis from seven processing plants. *Salmonella spp*. and *Photobacterium spp*. were found on salmon at the slaughter stage (44). The main microbiota of air-packaged (AP) and vacuum-packaged (VP) carp filets during storage were systematically identified by Zhang et al. (52) The results showed that *Pseudomonas aeruginosa* was the only microbiota found in spoiled AP carp, while *Karnococcus* were found mainly in VP samples (52). Characterization of some specific H_2_S-producing spoilage organisms isolated from raw tuna and swordfish by Serio et al. (45). Among them, *Shewanella spp*. can form biogenic amines, showing great corruption potential. *Pseudomonas* and *Shewanella* are two spoilage microorganisms of frozen fish meat preserved aerobically, while CO_2_-resistant *Photobacterium phosphoreum* is the main flora of fish meat packed under altered atmosphere conditions (46). *Pseudomonas* can inhibit each other in seafood matrices. Boziaris et al. (53) observed that *Pseudomonas fluorescens* outcompeted *Pseudomonas spp*. at increased storage temperatures and that *Pseudomonas spp*. could cause spoilage bacteria in raw salmon under aerobic conditions (53). *Brochothrix thermosphacta* produces caramel off-flavors (2,3-butanedione) in seafood under aerobic conditions. The genus *Psychrobacter* is a gram-negative, psychrophilic and aerobic bacterium found mainly in seafood and meat. Members of this category include *Acinetobacter, Photosynthetic bacteria* (*Psb*) *cibatius, Psb. maritimus* and *Psb. proteolyticus* are found in a variety of seafood, such as mackerel, anglerfish, lobster, oysters, and Atlantic cod (47). *Psychrobacter* species, especially *Psb. immobilis*, are able to break down lipids and hydrolyze amino acids, thus causing a slight ichthyological and musty odor.

## Common control approaches

### Packaging methods

Factors affecting the growth of microorganisms in meat include intrinsic factors (natural and added ingredients, pH, redox potential, and water activity), as well as extrinsic factors (storage temperature and packaging methods).

Modified atmospheric packaging (MAP) reported extending the shelf life of frozen meat (54). Luong et al. (55) showed that for fresh turkey sausage, a 2% (w/w) lactic acid formulation in combination with MAP (50% CO_2_-50% N_2_) significantly reduced acidification, off-flavors and prevented discoloration of the sausage from red to dark gray or brown. In pork sausages, MAP (70% O_2_-30% CO_2_) slightly reduces off-flavor perception (55). The decrease in the quality of meat during storage depends not only on the number of bacteria but also on the activity of bacterial metabolism. Proteases produce free amino acids which can be further metabolized by bacteria, resulting in off-flavors and mucus associated with spoilage. Previous studies have shown that a gas mixture of 30% CO_2_ and 70% N_2_ for MAP can extend the shelf life of frozen chicken (56). Meat stored under this MAP has a lower number of *Pseudomonas spp*. and is less likely to spoil than when stored in the air (57). Therefore, MAP may affect the growth as well as the metabolism of bacteria. Different packaging conditions affect the shelf life of carp and the growth of microorganisms. The shelf life of air-packed (AP) and vacuum-packed (VP) filets at 4°C is 8 days and 12 days, respectively, with the highest number of *Pseudomonas aeruginosa* in the AP sample and a relatively high level of lactic acid bacteria (LAB) in the VP sample. VP delays the increase in biogenic amine content compared to AP (52).

Dohlen et al. (58) studied the effect of novel antimicrobial packaging materials containing poly-[2-(tertbutylamino) methylstyrene] (poly-TBAMS) on the growth of typical spoilage and pathogenic bacteria present in meat. The results showed that gram-positive bacteria were more susceptible to poly(TBAMS) foil than gram-negative bacteria, and an increase of the antimicrobial activity with an increasing amount of poly(TBAMS) in the base polymer (58). Amna et al. (59) developed a new antimicrobial hybrid packaging pad consisting of biodegradable polyurethane. This type of packaging material was found to show effective antibacterial activity against *Staphylococcus aureus* and *Salmonella typhimurium* (59). Zeinab et al. (60) found that TiO_2_ nanocomposites and irradiation at 3kGy maintained chemical, microbiological, and sensory properties for longer periods and extended the shelf life of fish filets in cold storage (60). It was shown that antibacterial polyvinyl alcohol films containing TiO_2_ nanoparticles inhibited *Shewanella spp*., *Pseudomonas putida*, and *Aeromonas hydrophila*, and prolonged the shelf life of macroscopic rotenone by 1–2 days (61).

### Addition of antibacterial substances

Recently, the harmful effects associated with synthetic preservatives have led to a search for new alternatives in natural products. Commercially available polyphenols reduce primary and secondary lipid peroxidation levels, inhibit lipoxygenase activity, improve meat color stability, minimize degradation of salt-soluble myogenic fibrin and sulfhydryl groups, and retard bacterial growth (62). Essential oils (EOs) are secondary metabolites obtained from plants of Asteraceae, Lamiaceae, Lauraceae, Myrtaceae, Rutaceae, Umbelliferae, Zingiberaceae families, among others. Composed of a complex mixture of low molecular weight volatile compounds (63). These valuable substances can be obtained from different parts of the plant, such as bark, flowers, fruits, leaves, roots, and stems (64). This section summarizes the effects of common plant EOs on spoilage microorganisms in meat products and fish meat (Table 3).

**Table 3:** List of common plant EOs effects on spoilage bacteria in meat products and fish meat.

**NO**.	**EOs**	**Meat types**	**Effects**	**References**
1	Sage EO	Fresh pork sausages	Inhibiting thermophilic aerobic bacterial.	(65)
2	*Satureja montana L*. EO	Fresh pork sausages	Reducing the number of thermophilic aerobic bacteria and *Enterobacteriaceae*.	(66)
3	*Z.clinopodioides* EO+nisin	Raw beef patty	Inhibiting the growth of Cryophilic, *Enterobacteriaceae*, and thermophilic bacteria as well as *Staphylococcus aureus* and *Escherichia coli* O157:H7.	(67)
4	Rosemary, sage, thyme, and clove Eos	Smoked rainbow trout	Extending the shelf life by 6-7 weeks.	(68)
5	Black pepper EO	Fresh pork	Deformation, depression, shrinkage, adhesion, and rupture of *Escherichia coli*; Inhibiting the growth of *Pseudomonas* and *Enterobacteriaceae*.	(69, 70)
6	Cinnamon EO	Fresh Italian style sausages	Inhibiting the growth of *Enterobacteriaceae*.	(71)

Thymol and carvacrol have inhibitory effects on *Bacillus cereus, Pseudomonas aeruginosa*, and *Staphylococcus aureus* (72). In contrast, Guimarães et al. (73) observed that the free terpenes commonly found in essential oils have strong antibacterial activity against gram-negative bacteria (73). Sage EO as a preservative was demonstrated in meat products used as fresh pork sausages, suppressing aerobic thermophilic bacterial counts at the end of storage (4.8–7.3%) (65). Sojic et al. (66) evaluated the antibacterial potential of *Satureja montana L*. EO in fresh pork sausage. Compared with the control group, adding *Satureya montana L*.EO can improve the microbial stability of the product, and reduce the total number of thermophilic aerobic bacteria (4.9-10.9%) and *Enterobacteriaceae* (7.1-19.6%) in sausage (66). Shahbazi et al. (67) found that both essential oils and lactobacillus peptides significantly (*p* < 0.05) affected the growth of Cryophilic, *Enterobacteriaceae*, and thermophilic bacteria as well as *Staphylococcus aureus* and *Escherichia coli* O157:H7 in raw beef patties, with the fastest decrease in the number of tested microorganisms in samples treated with 0.2% essential oil + 500 IU/g lactobacillus peptide (67).

For fish meat, MAP conditions favor the growth of anaerobic bacteria, which can produce toxins. Therefore, often in combination with other modalities (MAP, edible coatings and films, non-thermal sterilization, etc.) to enhance the effectiveness of natural preservatives (74). Yuan et al. (75) found that the total volatile alkaline nitrogen and total aerobic colony values of black spot shrimp treated with chitosan coating in combination with pomegranate peel extract (PPE) were lower than those of shrimp treated with chitosan coating or PPE alone, indicating a synergistic effect between chitosan coating and PPE (75). Emird et al. (68) found that rosemary, sage, thyme, and clove essential oils as natural antioxidants can be used with vacuum packaging to extend the shelf life of smoked rainbow trout by 6–7 weeks (68). In a previous study, the effects of nanoemulsions based on commercial oils (sunflower, canola, corn, olive, soybean, and hazelnut oils) on the fatty acid compositions of farmed sea bass stored at 2 ± 2°C was investigated. The results showed hazelnut group gave the highest polyunsaturated fatty acid content, followed by canola and soybean at the end of the storage period. These oils can be recommended for nanoemulsions as a preservative for fish (76).

### Plasma sterilization

Reactive oxygen species (ROS) in atmospheric pressure cold plasma (APCP) act on gram-positive and gram-negative bacteria through different microbicidal mechanisms, and intracellular ROS levels increase in *Listeria monocytogenes* and *Staphylococcus aureus* with prolonged exposure to APCP, but with little damage to the cell wall (77). Exposure of *Listeria monocytogenes* and *Staphylococcus aureus* to APCP causes cell shrinkage, but little damage to the cell wall. In addition, intracellular ROS levels of *Listeria monocytogenes* and *Staphylococcus aureus* have been shown to increase with the exposure time of APCP (78). Dielectric barrier discharge (DBD) plasma is a source of plasma that generates ROS that can penetrate cell membranes and cause apoptosis through intracellular DNA damage. Previous studies have shown that the levels of *Listeria monocytogenes* in inoculated meat and meat products were reduced by 0.59–6.52 Log CFU/g after DBD treatment (79).

### Bacteriophage sterilization

Phages are considered promising new bioretention agents because they can efficiently and specifically lyse targeted bacteria. A cocktail of three phages effectively inhibited the growth of *S. hiva* in catfish filets and significantly improved the pH, total volatile basic nitrogen, and organoleptic value indices of the filets (80).

### Low-dose irradiation

Low-dose irradiation is considered a common technique for keeping fish meat fresh. Dogruyol et al. reported that sous-vide filets could be irradiated (5.0 kGy) to extend their shelf life up to 8 weeks during refrigerated storage without any damage to the organoleptic and physicochemical properties of the filets (81).

## Conclusion

Microbial contamination of meat is the domain cause of losses during production, storage, and distribution, accounting for approximately 21% of total food losses. We review recent advances in research on microbial diversity causing spoilage of livestock, poultry, and fish meat and summarize measures to prevent MS. However, to achieve more accurate and effective control of spoilage microorganisms in meat, it is necessary to obtain more comprehensive and accurate information on the composition of microbial communities and the dynamic processes of their metabolism. By revealing specific interactions between various spoilage phenotypes during MS, we would achieve controllable product quality during the production, transportation, marketing, and storage of meat.

## Author contributions

YZ and LC conceived and wrote the original draft. WW, LC, ML, JZ, RZ, LJ, ZZ, and DC reviewed, edited, and revised the manuscript. All authors approved the finalversion.

## Funding

This work was supported by the National Modern Agricultural Industrial Technology System, Sichuan Innovation Team Construction Project (SCSZTD-2022-08-07), Liangshan Science and Technology Program (22ZDYF0249, 21CGZH0001), and the Natural Science Foundation of Jiangsu Province (BK20200932).

## Conflict of interest

The authors declare that the research was conducted in the absence of any commercial or financial relationships that could be construed as a potential conflict of interest.

## Publisher's note

All claims expressed in this article are solely those of the authors and do not necessarily represent those of their affiliated organizations, or those of the publisher, the editors and the reviewers. Any product that may be evaluated in this article, or claim that may be made by its manufacturer, is not guaranteed or endorsed by the publisher.
